# Association Between Kawasaki Disease and Childhood Epilepsy: A Nationwide Cohort Study in Taiwan

**DOI:** 10.3389/fneur.2021.627712

**Published:** 2021-04-06

**Authors:** Chien-Heng Lin, Jung-Nien Lai, Inn-Chi Lee, I-Ching Chou, Wei-De Lin, Mei-Chen Lin, Syuan-Yu Hong

**Affiliations:** ^1^Division of Pediatrics Pulmonology, China Medical University, Children's Hospital, Taichung, Taiwan; ^2^Department of Biomedical Imaging and Radiological Science, College of Medicine, China Medical University, Taichung, Taiwan; ^3^School of Chinese Medicine, College of Chinese Medicine, China Medical University, Taichung, Taiwan; ^4^Department of Chinese Medicine, China Medical University Hospital, Taichung, Taiwan; ^5^Department of Pediatrics, School of Medicine, Chung Shan Medical University Hospital and Institute of Medicine, Chung Shan Medical University, Taichung, Taiwan; ^6^Division of Pediatrics Neurology, China Medical University, Children's Hospital, Taichung, Taiwan; ^7^College of Chinese Medicine, Graduate Institute of Integrated Medicine, China Medical University, Taichung, Taiwan; ^8^Department of Medical Research, China Medical University Hospital, Taichung, Taiwan; ^9^Management Office for Health Data, China Medical University Hospital, Taichung, Taiwan; ^10^College of Medicine, China Medical University, Taichung, Taiwan

**Keywords:** Kawasaki disease, children, epilepsy, seizure, risk

## Abstract

**Background:** Kawasaki disease is a common vasculitis of childhood in East Asia. The complications following Kawasaki disease mostly included cardiovascular sequelae; non-cardiac complications have been reported but less studied. This study investigated potential epilepsy following Kawasaki disease in Taiwanese children.

**Objectives:** Through National Health Insurance Research Database, we retrospectively analyzed the data of children aged <18 years with clinically diagnosed Kawasaki disease from January 1, 2000 to December 31, 2012 in Taiwan. These patients were followed up to estimate the incidence of epilepsy in the Kawasaki cohort in comparison with that in the non-Kawasaki cohort in Taiwan.

**Results:** A total of 8,463 and 33,872 patients in the Kawasaki and non-Kawasaki cohorts were included in the study, respectively. Of the total eligible study subjects, 61.1% were boys and 38.9% were girls; most patients with newly diagnosed Kawasaki disease were aged <5 years [88.1%]. Patients with Kawasaki disease showed a higher incidence rate [47.98 vs. 27.45 every 100,000 person years] and significantly higher risk [adjusted hazard ratio = 1.66, 95% confidence interval = 1.13–2.44] of epilepsy than those without the disease. Additionally, female sex [adjusted hazard ratio = 2.30, 95% confidence interval = 1.31–4.04] and age <5 years [adjusted hazard ratio = 1.82, 95% confidence interval = 1.22–2.72] showed a significantly higher risk of epilepsy in the Kawasaki cohort.

**Conclusion:** Results revealed a higher incidence rate and significant risk of epilepsy in Taiwanese children with Kawasaki disease than in those without the disease. Therefore, children diagnosed with Kawasaki disease are recommended follow-up as they have a high risk of epilepsy and seizure disorders.

## Background

Kawasaki disease [KWD], also known as mucocutaneous lymph node syndrome, has long been considered a common childhood vasculitis disorder with various cardiovascular complications, most commonly involving medium-sized muscular arteries (1). Additionally, non-cardiac complications followed by KWD has aroused interest during the past few decades (2). Despite neurological complications involving the central nervous system (CNS) not being uncommon in KWD, the extent of their influence on the brains of children and the effect of KWD on the development of various neurodevelopmental disorders (NDD) remains unclear (3). Although neurological complications such as seizures, hemiplegia, facial palsy, cerebral vasculitis, myositis, and aseptic meningitis have been occasionally reported in conjunction with KWD, the long-term sequelae such as epilepsy have never been reported (4–7).

In precedence to the present study, our research team conducted a study in 2019 that included 612 children with KWD. The results of a long-term follow-up indicated that these children had an increased risk of developing a neurodevelopmental disorder (NDD) with KWD. Although not all of the children developed an NDD, the prevalence was statistically significant for epilepsy and Tourette syndrome (8). Here, we conducted a wide-ranging investigation in the child population by using Taiwan National Health Insurance Research Database [NHIRD] to determine whether an association exists between KWD and childhood epilepsy.

## Methods

### Data Source

National Health Insurance program [Taiwan NHI] was established in 1995 and enrolled >99.9% of the population in Taiwan. NHIRD was established by Taiwan NHI and consisted of information on outpatients, hospitalization, medical treatment or operation, and other medical services of patients' each hospital visit. To conduct this study analysis, we used a population-based inpatient file based on NHIRD. The identification number was encrypted before the database was released to protect the privacy of each study subject. Diagnoses in Taiwan NHI are coded according to the International Classification of Disease, Ninth Revision, Clinical Modification [ICD-9-CM]. The Research Ethics Committee of China Medical University and Hospital in Taiwan approved the study [CMUH-104-REC2-115-R4].

### Study Population

**Figure 1:**
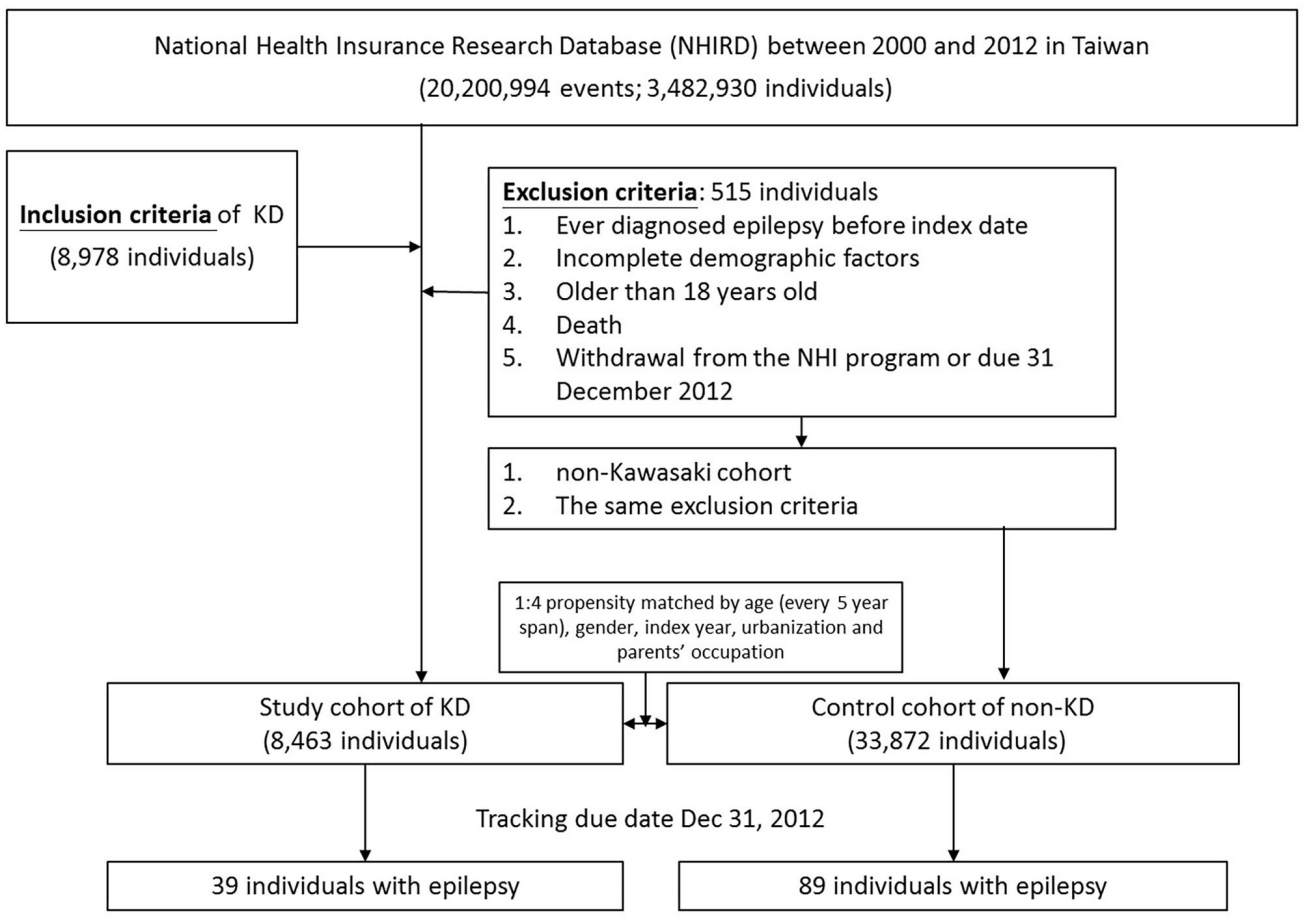
The flowchart of study population selection.

To clarify the association between KWD [ICD-9-CM: 446.1] and epilepsy [ICD-9-CM: 345, A225], we identified two cohorts: Kawasaki and non-Kawasaki cohorts. Patients who were newly diagnosed as having KWD from 2000 to 2012 were classified into the Kawasaki cohort, and the diagnosis date was set as the index date; patients without any KWD diagnosis were classified into the non-Kawasaki cohort. Patients ever diagnosed as having epilepsy before the index date, with incomplete demographic factors, >18 years old, who died, or who withdrew from the NHI program or due date to December 31, 2012 were excluded from this study. Each patient in the Kawasaki cohort was 1:4 propensity matched based on age [every 5-year span], sex, index year, urbanization, and parent occupation (Figure 1). To avoid wrongly diagnosing acute seizures as epilepsy during the KWD course, the gain of diagnosis with epilepsy was separated from the admission course of KWD for at least 1 month.

### Statistical Analysis

We used two sample *t*-tests for age and chi-square test for sex, urbanization, and parent occupation to compare the difference between Kawasaki and non-Kawasaki cohorts. The Kaplan–Meier method was used to demonstrate the cumulative incidence of epilepsy and cerebrovascular diseases between two cohorts, and the log rank test was applied to estimate the difference between the two curves.

To calculate epilepsy risk between Kawasaki and the comparison cohorts, we used a Cox proportional-hazard model and presented hazard ratios, adjusted hazard ratios [aHRs], and 95% confidence intervals [CIs] before and after adjusting demographic factors. The significance criterion was set at <0.05 for two-side testing of *p-*value. All statistical analyses were performed using SAS statistical software, version 9.4 [SAS Institute Inc., Cary, NC, USA]; the cumulative incidence curve was plotted using R software.

## Results

**Figure 2:**
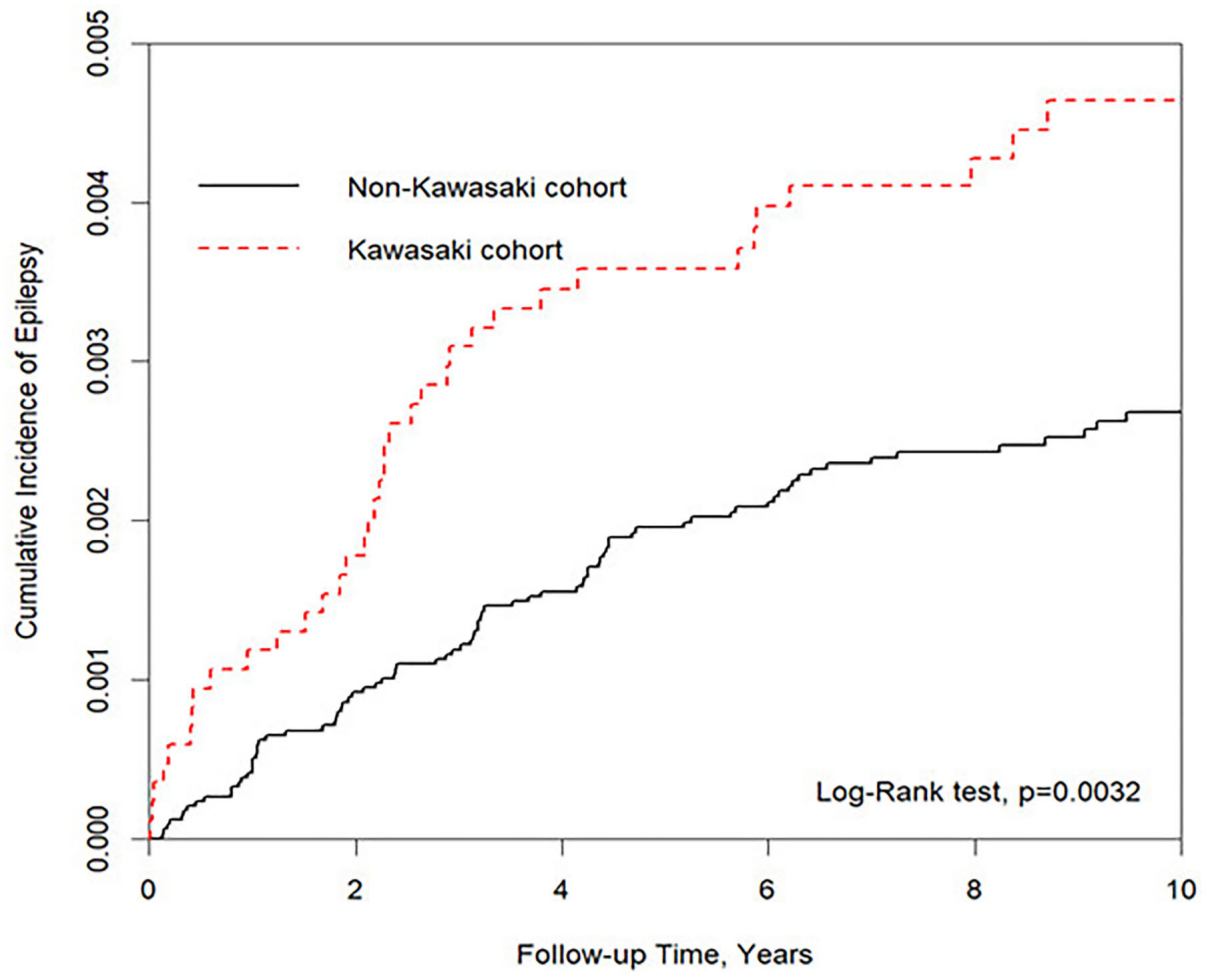
Kaplan–Meier for cumulative incidence of epilepsy under aged stratified by Kawasaki disease with log-rank test.

We eventually enrolled 8,463 and 33,872 patients in the Kawasaki and non-Kawasaki cohorts. There were no statistically significant differences in urbanization level, geographic region, head injury, parents' occupation (all *p* > 0.05) (Table 1), respectively. Of the total eligible study subjects, 61.1% were boys and the other 38.9% were girls; most patients were newly diagnosed as having KWD at age ≤5 years [88.1%], and the mean age was 2.4 years. Table 2 presents the number, incidence rate, and epilepsy risk in the Kawasaki and non-Kawasaki cohorts. Patients with KWD showed a higher incidence rate [47.98 vs. 27.45 every 100,000 person years] and significantly higher risk [aHR = 1.66, 95% CI = 1.13–2.44] of epilepsy. Other potential risk factors such as sex, increasing age, and urbanization had a non-significant effect on epilepsy risk. To confirm the association between KWD and epilepsy, a multivariable stratified analysis was performed. Female sex [aHR = 2.30, 95% CI = 1.31–4.04] and age <5 years [aHR = 1.82, 95% CI = 1.22–2.72] showed a significantly higher risk of epilepsy in the Kawasaki cohort (Table 3). After stratification, patients with a follow-up period of 5 years after KWD diagnosis had a significantly higher risk of epilepsy [aHR = 1.82, 95% CI = 1.17–2.82] than those with a follow-up period of >5 years (Table 4). Patients with KWD had a significantly higher cumulative incidence of epilepsy [*p* = 0.0032] than did those without KWD (Figure 2).

**Table 1:** Demographic characteristics of patients with Kawasaki disease in Taiwan during 2000–2012.

**Variable**	**Children without Kawasaki disease**	**Children with Kawasaki disease**	***p*-value**
	***n =* 33,872**	***n =* 8,463**	
	n (%)/mean (SD)^*^^†^	n (%)/mean (SD)^*^^†^	
**Gender**			0.976
Female	13,168 (38.9)	3,292 (38.9)	
Male	20,704 (61.1)	5,171 (61.1)	
**Age at baseline**			1.000
<5	29,826 (88.1)	7,454 (88.1)	
5–10	3,521 (10.4)	879 (10.4)	
10–18	525 (1.5)	130 (1.5)	
Mean(SD)	3.4 (2.4)	2.4 (2.3)	
**Urbanization^§^**			0.997
1 (highest)	10,224 (30.2)	2,556 (30.2)	
2	10,068 (29.7)	2,517 (29.7)	
3	13,580 (40.1)	3,390 (40)	
**Occupation**			0.977
Office workers	22,549 (66.6)	5,626 (66.5)	
Manual workers	7,185 (21.2)	1,804 (21.3)	
Others	4,138 (12.2)	1,033 (12.2)	

* *Chi-square test, Student's t-test*.

† *SD, standard deviation*.

§ *The NHRI stratified all city districts and townships in Taiwan into 7 urbanization levels, based on population density (people/km2), proportion of residents with higher education, elderly and agricultural population, and the number of physicians per 100,000 people in each area. Level 1 represented areas with a higher population density and socioeconomic status, and level 7 represented the lowest. Because few people lived in more rural areas of levels 3–7, our study grouped these areas into the level 3 group. Referenced from Liu et al. (Incorporating development stratification of Taiwan townships into sampling design of large scale health interview survey. J Health Manag 2006; 14: 122)*.

**Table 2:** Cox model measured hazard ratio and 95% confidence intervals of epilepsy associated with and without Kawasaki disease.

**Characteristics**	**Epilepsy event**	**Person**	**IR**	**Univariable**		**Mutivariable**	
	**(*n =* 128)**	**years**		**HR (95% CI)**	***p-*value**	**HR (95% CI)**	***p-*value**
**Kawasaki disease**							
No	89	324,265	27.45	Ref.		Ref.	
Yes	39	81,276	47.98	1.75 (1.20–2.55)	0.004	1.66 (1.13–2.44)	0.009
**Gender**							
Female	54	157,028	34.39	Ref.		Ref.	
Male	74	248,513	29.78	0.87 (0.61–1.23)	0.431	0.87 (0.61–1.24)	0.435
**Age at baseline**							
<5	112	362,960	30.86	Ref.		Ref.	
5–10	14	36,681	38.17	1.18 (0.68–2.06)	0.555	1.19 (0.68–2.08)	0.536
10–18	2	5,901	33.89	1.08 (0.27–4.38)	0.913	1.07 (0.27–4.35)	0.920

*HR, hazard ratio; CI, confidence interval; IR, incidence rates, per 100,000 person-years.

**Adjusted HR: adjusted for gender, age, urbanization, and occupation in Cox proportional hazards regression.

**Table 3:** Incidence rates, hazard ratio and confidence intervals of epilepsy in different stratification.

**Variables**	**Non-Kawasaki cohort**			**Kawasaki cohort**			**Compared to non-Kawasaki cohort**			
	***n =* 33,872**	***n =* 8,463**	**Crude HR**	***p-*value**	**Adjusted HR**	***p-*value**	**(95% CI)**		**(95% CI)**	
	**Event**	**Person years**	**IR**	**Event**	**Person years**	**IR**				
**Epilepsy**										
**Gender**										
Female	34	125,545	27.08	20	31,483	63.53	2.35 (1.35–4.08)	0.002	2.30 (1.31–4.04)	0.004
Male	55	198,720	27.68	19	49,793	38.16	1.38 (0.82–2.33)	0.226	1.28 (0.75–2.18)	0.363
**Age at baseline**										
<5	77	290,289	26.53	35	72,671	48.16	1.82 (1.22–2.71)	0.003	1.82 (1.22–2.72)	0.003
5–10	10	29,299	34.13	4	7,382	54.19	1.60 (0.50–5.09)	0.429	1.60 (0.51–5.09)	0.429
10–18	2	4,677	42.76	0	1,224	0.00	–	–	–	–

*IR, incidence rates, per 100,000 person-years; HR, hazard ratio; CI, confidence interval.

**Adjusted HR: adjusted for gender, age, urbanization, and occupation in Cox proportional hazards regression.

**Table 4:** Incidence rates, hazard ratio, and confidence intervals of epilepsy in different follow-up stratification.

**Follow-up years**	**Non-Kawasaki cohort**			**Kawasaki cohort**			**Compared to non-Kawasaki cohort**			
	***n =* 33,872**	***n =* 8,463**	**Crude HR**	***p-*value**	**Adjusted HR**	***p-*value**	**(95% CI)**		**(95% CI)**	
	**Event**	**Person years**	**IR**	**Event**	**Person years**	**IR**				
**Epilepsy**										
<5	65	165,594	39.25	30	41,424	72.42	1.85 (1.20–2.84)	0.006	1.82 (1.17–2.82)	0.008
≥5	24	158,671	15.13	9	39,852	22.58	1.49 (0.69–3.21)	0.305	1.24 (0.57–2.71)	0.590

*IR, incidence rates, per 100,000 person-years; HR, hazard ratio; CI, confidence interval.

**Adjusted HR: adjusted for gender, age, urbanization, and occupation in Cox proportional hazards regression.

## Discussion

As early as 1977, Nakane et al. reported a case of mucocutaneous lymph node syndrome complicated by acute occlusion of the right middle cerebral artery and hemiconvulsion–hemiplegia–epilepsy syndrome. Since then, numerous neurological involvements associated with KWD have been discovered (9). These include headache, convulsions, somnolence, irritability, meningoencephalit5is, ptosis, sensorineural hearing loss, and facial palsy (4, 6, 7). Even so, most of them subsided completely following the successful management of KWD without any clinically apparent signs of CNS injury. However, few studies have examined whether acute KWD is followed by subtle but clinically apparent changes in the brain of children, which might lead to the long-term development of NDDs (4–7). Therefore, the present study adopted a larger nationwide database to trace the incidence of epilepsy in Taiwanese children with KWD. In the present study, we demonstrated that children with KWD experience epilepsy later in life. The trend was independent of sex, age at KWD diagnosis, and urbanization.

Additionally, our results revealed that children aged <5 years with KWD had a higher risk of epilepsy. We hypothesize that from birth to age 5 years, a child's brain develops more than at any other time in life (10), and KWD, a vasculitis that occurs most often in early childhood, may interfere with brain development and probably subtly alter the structure of human brain, causing epilepsy (11). Although no reports refer to alterations of the brain structure of children following KWD, and our cohort observation study failed to provide evidence of such alterations, we presumed the involvement of a mechanism and pathogenesis comparable to those of central nervous system vasculitis, in which brain vessel wall inflammation can result in intraluminal stenosis and platelet adhesion to the vessel wall, eventually leading to a substantial brain parenchymal lesion and the development of seizure disorders (12, 13). We also determined that epileptogenesis largely occurred within 5 years of KWD diagnosis. Although no existing reports explain it, we speculated that such would be the case, with time for secondary injury processes (such as neuroinflammation) following KWD to occur (14, 15). A similar phenomenon occurs in epileptogenesis after traumatic brain injury (16). However, more evidence derived from neuroimaging studies and animal brain models is necessary to validate our hypothesis.

This study revealed that female patients with KWD had a higher risk of epilepsy than did male patients, but the reason underlying these finding remains unclear. One argument is that women have stronger immune responses to infection and inflammation than men do (17). In addition, sex hormones play a crucial role in immune system activities (18). Women undergo endocrinological changes two to three times in their lifetime [puberty, childbearing, breastfeeding, and menopause], and each hormone's transition results in a significant effect on the immune system, rendering female patients with an underlying autoimmune disease susceptible to associated complications (19, 20). This theory was studied in systemic lupus erythematosus—a well-known autoimmune disease (21). However, because the present study's patients were young children (due to the nature of KWD), the aforementioned assumption regarding endocrinological changes, which is suitable for people who are adolescents or older, may not be applicable here. Perhaps hormones present only in boys (e.g., testosterone) played a role in our results. Because testosterone has been proven to play roles in the inhibition of vascular inflammation, neuroinflammation, and aneurysm formation (22–26), its possible role in the inhibition of brain inflammation associated with KWD would be another area worth studying.

Nonetheless, previous studies have suggested that early brain insult might alter the brains of boys and girls differently but lead to the development of NDDs later in life (27). For example, autism spectrum disorder and attention-deficit/hyperactivity disorder are more likely to be diagnosed in boys (28, 29). A study in the rat model, using lipopolysaccharide as a stimulator of the innate immune system, examined blood–brain barrier dysfunction under inflammatory conditions. It was found that following lipopolysaccharide stimulation, females had higher levels of cytokines and chemokines in the blood and brain compared with males. Hence, although females have a stronger capability to resist infections, females may have an exacerbated inflammatory response compared with males when presented with the same dose of an immune stimulus (30). Further research is required to confirm whether the same theory can be applied in female patients with epilepsy following KWD.

In addition to epilepsy, some long-term behavioral problems, including somatic problems and withdrawal trait, have been noted in children following KWD (Table 5) (8, 31–37), but the conclusions are conflicting (37). Separately, the role of brain-reactive autoantibodies has been studied in brain development and cognitive impairment from many different autoimmune conditions and autoimmune encephalitis (38–41), Likewise, some autoantibodies were studied in relation to KWD, including antithyroid microsomal antibody, antiparietal cell antibody, antiliver kidney microsomal antibody, antiendothelial cell antibodies, antinuclear antibody, antineutrophil cytoplasmic antibody, antimitochondrial antibody, and antismooth muscle antibody. These autoantibodies likely develop in children with KWD during the acute stage and may persist for many years (42, 43). However, whether these autoantibodies increase the risk of autoimmune disease in the future is unclear, although some studies have explored the association between them (44, 45). Furthermore, epilepsy after KWD is another problem, and whether epilepsy after KWD shares a common mechanism as seizure occurring in the acute stage of vasculitis during KWD or any other autoimmune encephalitis remains unclear and intrigues us. If such is the case, two hypotheses may be explained. First, some autoantibodies triggered by KWD could persist *in vivo* for years, which in turn leads to some autoantibody-mediated diseases or systemic autoimmune diseases so that seizure disorders or epilepsy could only be sentinel disorders of these diseases (46, 47). Second, given that medium-sized vessel vasculitis is the bedrock of KWD, it is reasonable to infer that human brain, an organ rich in vessels, is subject to cryptogenic changes in structures, which contribute to epilepsy later in life (48–50). Although none has been proven, both are possible. Future studies are encouraged to investigate these assumptions.

**Table 5:** Literature search and article analysis for neurodevelopmental disorders in children with KWD.

**Study [reference]**	**Research type**	**Case numbers of KWD**	**Long term F/U**	**Comments**
Carlton-Conway et al. (31)	Case control	65	Yes	KWD can be associated with significant behavioral sequelae. Long-term follow up and referral to a clinical psychologist may be necessary in these patients
Baker et al. (32)	Retrospective	110	Yes	KWD patients without coronary artery aneurysms were similar to the general population in their general physical and psychosocial health
King et al. (33)	Cohort analytic	32	Yes	KWD poses no effect on childhood cognitive development and academic performance
Nishad et al. (34)	Case control	20	No	No significant difference in the three assessments of social adaptation, IQ, and behavioral functioning of children with KWD
Kuo et al. (35)	Retrospective cohort	563	Yes	Patients with KWD are not at increased risk of ASD
Kuo et al. (36)	Retrospective cohort	651	Yes	Patients with KWD may not have an increased risk of ADHD, however, the real association between KWD and ADHD remains unclear
Wang et al. (37)	Case control and retrospective cohort	168 in clinical settings; 4,286 in nationwide cohort	Yes	KWD does not increase a child's risk of future cognitive impairment in both the clinical data and the population-based cohort
Lin et al. (8)	Retrospective cohort	612	Yes	KWD is associated with heterogeneous NDDs and is significant in the prevalance of TS and epilepsy

*ADHD, attention deficit hyperactivity disorder; ASD, Autism spectrum disorder; F/U, follow-up; IQ, Intelligence Quotient; KWD, Kawasaki disease; TS, Tourette syndrome*.

This study has some limitations. First, we were unable to preclude some confounding factors, including pharmacological treatment of children with KWD, KWD severities, and other genetic and environmental risk factors associated with KWD. Second, the diagnoses of epilepsy and KWD in this study were derived based on the ICD-9-CM code. Although it is a powerful tool for disease categorization, it lacks scientific validation. Third, although a retrospective cohort study is an effective approach to enrolling numerous individuals and discovering hidden medical phenomenon over a long time period and from a broad perspective, the approach cannot explore underlying mechanisms or pathogeneses of diseases. Hence, this study might serve as a pilot study attracting public attention and further research. The recommended methods are (1) randomized control trials to provide more rigorous conclusions with appropriate control of confounders such as the pharmacological treatment of KWD and KWD severity; and (2) animal model–based studies to clarify the pathogenesis of epilepsy following KWD.

## Conclusions

The results of this study revealed that KWD in childhood might be a potential risk factor for childhood epilepsy. Therefore, the brain development of children diagnosed as having KWD should be carefully followed up using neuroimaging to monitor for the development of epilepsy, especially within the 5 years after KWD diagnosis. A massive randomized control trial and a detailed, structured animal cell model containing different cofounders are necessary in future.

## Data Availability Statement

The data analyzed in this study is subject to the following licenses/restrictions: The analysis was generated by the national health insurance database, which was safeguarded and was only accessed by a strict application process. Requests to access these datasets should be directed to Mei-Chen Lin, coolindm@gmail.com.

## Author Contributions

S-YH collected the data, analyzed the data, and prepared the initial draft of the manuscript. I-CL and C-HL took part in designing the study and wrote the final draft of the manuscript. The statistics for the study were compiled by J-NL, W-DL, M-CL, and I-CC, who also took part in the editing process and the revision of the tables. All of the authors read and approved of the final manuscript.

## Conflict of Interest

The authors declare that the research was conducted in the absence of any commercial or financial relationships that could be construed as a potential conflict of interest.

## References

[1] Sundel RP. Kawasaki disease. Rheum Dis Clin North Am. (2015) 41:63–73. 10.1016/j.rdc.2014.09.010 25399940

[2] Jindal AK Pilania RK Prithvi A Guleria S Singh S. Kawasaki disease: characteristics, diagnosis, and unusual presentations. Expert Rev Clin Immunol. (2019) 15:1089–104. 10.1080/1744666X.2019.1659726 31456443

[3] Falcini F. Kawasaki disease. Curr Opin Rheumatol. (2006) 18:33–8. 10.1097/01.bor.0000197998.50450.f6 16344617

[4] Liu X Zhou K Hua Y Wu M Liu L Shao S. Neurological involvement in Kawasaki disease: a retrospective study. Pediatr Rheumatol Online J. (2020) 18:61. 10.1186/s12969-020-00452-7 32664982 PMC7362431

[5] Martínez-Guzmán E Gámez-González LB Rivas-Larrauri F Sorcia-Ramírez G Yamazaki-Nakashimada M. Manifestaciones neurológicas en la enfermedad de Kawasaki atípica [Neurological manifestations in atypical Kawasaki disease]. Rev Alerg Mex. (2017) 64:376–80. 10.29262/ram.v64i3.231 29046034

[6] Zhang B Hao Y Zhang Y Yang N Li H Liang J. Kawasaki disease manifesting as bilateral facial nerve palsy and meningitis: a case report and literature review. J Int Med Res. (2019) 47:4014–8. 10.1177/0300060519854287 31364426 PMC6726819

[7] Hameed A Alshara H Schleussinger T. Ptosis as a complication of Kawasaki disease. BMJ Case Rep. (2017) 2017:bcr2017219687. 10.1136/bcr-2017-219687 PMC5534803 28601797

[8] Lin CH Lin WD Chou IC Lee IC Hong SY. Heterogeneous neurodevelopmental disorders in children with Kawasaki disease: what is new today? BMC Pediatr. (2019) 19:406. 10.1186/s12887-019-1786-y 31684911 PMC6827201

[9] Nakane A Yajima K Osawa M Fukuyama Y Kumagai K. A case of mucocutaneous lymph node syndrome complicated with acute occlusion of right middle cerebral artery and hemiconvulsions-hemiplegia-epilepsy syndrome (in Japanese). J Tokyo Wom Med Coli. (1977) 47:124–30.

[10] Gale CR O'Callaghan FJ Godfrey KM Law CM Martyn CN. Critical periods of brain growth and cognitive function in children. Brain. (2004) 127(Pt 2):321–9. 10.1093/brain/awh034 14645144

[11] Moharir M Shroff M Benseler SM. Childhood central nervous system vasculitis. Neuroimaging Clin N Am. (2013) 23:293–308. 10.1016/j.nic.2012.12.008 23608691

[12] Elbers J Halliday W Hawkins C Hutchinson C Benseler SM. Brain biopsy in children with primarain biopsy in children with primary small-vessel central nervous system vasculitis. Ann Neurol. (2010) 68:602–10. 10.1002/ana.22075 21031577

[13] Twilt M Benseler SM. Central nervous system vasculitis in adults and children. Handb Clin Neurol. (2016) 133:283–300. 10.1016/B978-0-444-63432-0.00016-5 27112683

[14] D'Ambrosio R Perucca E. Epilepsy after head injury. Curr Opin Neurol. (2004) 17:731–5. 10.1097/00019052-200412000-00014 15542983 PMC2672045

[15] Rana A Musto AE. The role of inflammation in the development of epilepsy. J Neuroinflammation. (2018) 15:144. 10.1186/s12974-018-1192-7 29764485 PMC5952578

[16] Webster KM Sun M Crack P O'Brien TJ Shultz SR Semple BD. Inflammation in epileptogenesis after traumatic brain injury. J Neuroinflammation. (2017) 14:10. 10.1186/s12974-016-0786-1 28086980 PMC5237206

[17] Klein SL Flanagan KL. Sex differences in immune responses. Nat Rev Immunol. (2016) 16:626–38. 10.1038/nri.2016.90 27546235

[18] Moulton VR. Sex hormones in acquired immunity and autoimmune disease. Front Immunol. (2018) 9:2279. 10.3389/fimmu.2018.02279 30337927 PMC6180207

[19] Desai MK Brinton RD. Autoimmune disease in women: endocrine transition and risk across the lifespan. Front Endocrinol. (2019) 10:265. 10.3389/fendo.2019.00265 31110493 PMC6501433

[20] Hughes GC. Progesterone and autoimmune disease. Autoimmun Rev. (2012) 11:A502–14. 10.1016/j.autrev.2011.12.003 22193289 PMC3431799

[21] Pan Q Chen X Liao S Chen X Zhao C Xu YZ. Updated advances of linking psychosocial factors and sex hormones with systemic lupus erythematosus susceptibility and development. PeerJ. (2019) 7:e7179. 10.7717/peerj.7179 31275761 PMC6598654

[22] Bianchi VE. The anti-inflammatory effects of testosterone. J Endocr Soc. (2018) 3:91–107. 10.1210/js.2018-00186 30582096 PMC6299269

[23] Vodo S Bechi N Petroni A Muscoli C Aloisi AM. Testosterone-induced effects on lipids and inflammation. Mediators Inflamm. (2013) 2013:183041. 10.1155/2013/183041 23606790 PMC3628213

[24] Mohamad NV Wong SK Wan Hasan WN Jolly JJ Nur-Farhana MF Ima-Nirwana S. The relationship between circulating testosterone and inflammatory cytokines in men. Aging Male. (2019) 22:129–40. 10.1080/13685538.2018.1482487 29925283

[25] Son BK Kojima T Ogawa S Akishita M. Testosterone inhibits aneurysm formation and vascular inflammation in male mice. J Endocrinol. (2019) 241:307–17. 10.1530/JOE-18-0646 31018175

[26] Kurth F Luders E Sicotte NL Gaser C Giesser BS Swerdloff RS. Neuroprotective effects of testosterone treatment in men with multiple sclerosis. Neuroimage Clin. (2014) 4:454–60. 10.1016/j.nicl.2014.03.001 24634831 PMC3952353

[27] From the American Association of Neurological Surgeons (AANS), American Society of Neuroradiology (ASNR), Cardiovascular Interventional Radiology Society of Europe (CIRSE), Canadian Interventional Radiology Association (CIRA), Congress of Neurological Surgeons (CNS), European Society of Minimally Invasive Neurological Therapy (ESMINT). Multisociety consensus quality improvement revised consensus statement for endovascular therapy of acute ischemic stroke. Int J Stroke. (2018) 13:612–32. 10.1177/1747493018778713 29786478

[28] Grissom NM Reyes TM. Let's call the whole thing off: evaluating gender and sex differences in executive function. Neuropsychopharmacology. (2019) 44:86–96. 10.1038/s41386-018-0179-5 30143781 PMC6235899

[29] Singh R Turner RC Nguyen L Motwani K Swatek M Lucke-Wold BP. Pediatric traumatic brain injury and autism: elucidating shared mechanisms. Behav Neurol. (2016) 2016:8781725. 10.1155/2016/8781725 28074078 PMC5198096

[30] Erickson MA Liang WS Fernandez EG Bullock KM Thysell JA Banks WA. Genetics and sex influence peripheral and central innate immune responses and blood-brain barrier integrity. PLoS ONE. (2018) 13:e0205769. 10.1371/journal.pone.0205769 30325961 PMC6191122

[31] Carlton-Conway D Ahluwalia R Henry L Michie C Wood L Tulloh R. Behaviour sequelae following acute Kawasaki disease. BMC Pediatr. (2005) 5:14. 10.1186/1471-2431-5-14 15916701 PMC1156909

[32] Baker AL Gauvreau K Newburger JW Sundel RP Fulton DR Jenkins KJ. Physical and psychosocial health in children who have had Kawasaki disease. Pediatrics. (2003) 111:579–83. 10.1542/peds.111.3.579 12612239

[33] King WJ Schlieper A Birdi N Cappelli M Korneluk Y Rowe PC. The effect of Kawasaki disease on cognition and behavior. Arch Pediatr Adolesc Med. (2000) 154:463–8. 10.1001/archpedi.154.5.463 10807296

[34] Nishad P Singh S Sidhu M Malhi P. Cognitive and behaviour assessment following Kawasaki disease–a study from North India. Rheumatol Int. (2010) 30:851–4. 10.1007/s00296-009-1078-1 19649637

[35] Kuo HC Wu CM Chang WP Kuo CN Yeter D Lin CY. Association between Kawasaki disease and autism: a population-based study in Taiwan. Int J Environ Res Public Health. (2014) 11:3705–16. 10.3390/ijerph110403705 24705358 PMC4025040

[36] Kuo HC Chang WC Wang LJ Li SC Chang WP. Association of Attention deficit hyperactivity disorder and Kawasaki disease: a nationwide population-based cohort study. Epidemiol Psychiatr Sci. (2016) 25:573–80. 10.1017/S2045796015000840 26392050 PMC7137668

[37] Wang LJ Kuo HC. Cognitive development after kawasaki disease- clinical study and validation using a nationwide population-based cohort. Circ J. (2018) 82:517–23. 10.1253/circj.CJ-17-0557 28890525

[38] Mader S Brimberg L Diamond B. The role of brain-reactive autoantibodies in brain pathology and cognitive impairment. Front Immunol. (2017) 8:1101. 10.3389/fimmu.2017.01101 28955334 PMC5601985

[39] Rubin DB Batra A Vaitkevicius H Vodopivec I. Autoimmune neurologic disorders. Am J Med. (2018) 131:226–36. 10.1016/j.amjmed.2017.10.033 29126826

[40] Dalmau J Graus F. Antibody-mediated encephalitis. N Engl J Med. (2018) 378:840–51. 10.1056/NEJMra1708712 29490181

[41] Hermetter C Fazekas F Hochmeister S. Systematic review: syndromes, early diagnosis, and treatment in autoimmune encephalitis. Front Neurol. (2018) 9:706. 10.3389/fneur.2018.00706 30233481 PMC6135049

[42] Basha A Rawat A Jindal AK Gupta A Anand S Garg R. Autoantibody profile in children with Kawasaki disease on long-term follow-up: a prospective study from North India. Int J Rheum Dis. (2018) 21:2036–40. 10.1111/1756-185X.13372 30168280

[43] Grunebaum E Blank M Cohen S Afek A Kopolovic J Meroni PL. The role of anti-endothelial cell antibodies in Kawasaki disease - *in vitro* and *in vivo* studies. Clin Exp Immunol. (2002) 130:233–40. 10.1046/j.1365-2249.2002.02000.x 12390310 PMC1906533

[44] Stagi S Simonini G Ricci L de Martino M Falcini F. Coeliac disease in patients with Kawasaki disease. Is there a link? Rheumatology. (2006) 45:847–50. 10.1093/rheumatology/kel007 16418194

[45] Sakurai Y. Autoimmune aspects of Kawasaki disease. J Investig Allergol Clin Immunol. (2019) 29:251–61. 10.18176/jiaci.0300 30183655

[46] Suurmond J Diamond B. Autoantibodies in systemic autoimmune diseases: specificity and pathogenicity. J Clin Invest. (2015) 125:2194–202. 10.1172/JCI78084 25938780 PMC4497746

[47] Ludwig RJ Vanhoorelbeke K Leypoldt F Kaya Z Bieber K McLachlan SM. Mechanisms of autoantibody-induced pathology. Front Immunol. (2017) 8:603. 10.3389/fimmu.2017.00603 28620373 PMC5449453

[48] Berlit P. Diagnosis and treatment of cerebral vasculitis. Ther Adv Neurol Disord. (2010) 3:29–42. 10.1177/1756285609347123 21180634 PMC3002614

[49] Garcia-Bermejo P Patro SN Ahmed AZ Al Rumaihi G Akhtar N Kamran S. Baseline occlusion angiographic appearance on mechanical thrombectomy suggests underlying etiology and outcome. Front Neurol. (2019) 10:499. 10.3389/fneur.2019.00499 31133981 PMC6517505

[50] Iannetti L Zito R Bruschi S Papetti L Ulgiati F Nicita F. Recent understanding on diagnosis and management of central nervous system vasculitis in children. Clin Dev Immunol. (2012) 2012:698327. 10.1155/2012/698327 23008735 PMC3447380

